# Acetylcholinesterase Inhibitors among *Zingiber officinale* Terpenes—Extraction Conditions and Thin Layer Chromatography-Based Bioautography Studies

**DOI:** 10.3390/molecules25071643

**Published:** 2020-04-03

**Authors:** Lidia Czernicka, Agnieszka Ludwiczuk, Edward Rój, Zbigniew Marzec, Agata Jarzab, Wirginia Kukula-Koch

**Affiliations:** 1 Chair and Department of Food and Nutrition, Medical University of Lublin, 4a Chodźki Str., 20-093 Lublin, Poland; lidia.czernicka@umlub.pl (L.C.); zbigniew.marzec@umlub.pl (Z.M.); 2 Independent Laboratory of Natural Products Chemistry, Chair and Department of Pharmacognosy, Medical University of Lublin; 1 Chodzki Str., 20-093 Lublin, Poland; aludwiczuk@pharmacognosy.org; 3 Supercritical Extraction Department, ŁUKASIEWICZ Research Network—New Chemical Syntheses Institute, Tysiąclecia Państwa Polskiego Ave. 13a, 24-110 Puławy, Poland; edward.roj@ins.pulawy.pl; 4 Department of Biochemistry and Molecular Biology, Medical University of Lublin, 1 Chodzki St., 20-093 Lublin, Poland; agata.jarzab@umlub.pl; 5 Chair and Department of Pharmacognosy, Medical University of Lublin; 1 Chodzki Str., 20-093 Lublin, Poland

**Keywords:** *Zingiber officinale*, Zingiberaceae, terpenes, neurodegeneration, acetylcholinesterase inhibitors, TLC bioautography, thin layer chromatography-high-performance liquid chromatography-mass spectrometry, gas chromatography-mass spectrometry, quantitative analysis, extraction optimization

## Abstract

Although numerous studies have been conducted on ginger extracts and fractions, the data on the pharmacological activity of single constituents of *Zingiber officinale* are still insufficient. To assess the antidementia properties of the plant, a thin layer chromatography (TLC)-based bioautography acetylcholinesterase inhibitory assay was performed on the *Zingiber officinale* diethyl ether extract. It led to the recognition of three active inhibitors among volatile constituents of the plant: *ar*-curcumene (A), *α*-sesquiphellandrene (B) and *a-*zingiberene (C). The identification of the components was possible thanks to the application of a TLC–HPLC-MS interface analysis of active zones and the GC-MS qualitative analysis of the tested samples. Based on the obtained results, the influence of several extraction techniques (hydrodistillation—HD, pressurized liquid extraction or accelerated solvent extraction—ASE, shaking maceration–SM, supercritical fluid extraction–SFE, and ultrasound-assisted extraction—UAE) on the recovery of the active metabolites from plant material was assessed to deliver enriched extracts. As a result, HD and SFE, were found to be the most efficient methods to recover the volatile components and the concentrations of A, B, and C reached 0.51 ± 0.025, 0.77 ± 0.045, and 1.67 ± 0.11 percent, respectively. Only HD and SFE were found to recover monoterpene hydrocarbons from the plant matrix. The remaining techniques provided extracts rich in more complex constituents, like sesquiterpenes.

## 1. Introduction

The occurrence of dementia and neurodegenerative diseases (like Alzheimer’s disease) is increasing, especially among the older population. It is estimated, that by 2050 ca. 115.4 million people will be affected by the progression of the disease. This fact has a significant impact on the budget of healthcare and social care around the world, especially because there is no effective drug against the development of cognitive disorders. The currently available medications can slow down the progression of dementia, but not to treat it. Based on the above information, the search for new potential drug candidates that retard the progression of memory impairment is of the highest importance [1,2]. Among the mechanisms of dementia are disturbances in acetylcholine synthesis and an overproduction of esterases, that decompose this neuromodulator, have been highlighted in various scientific reports. Acetylcholine has a crucial role in the supporting neuronal signal transmission, which helps to remember and recall events and things. Cognitive dysfunctions are observed, when acetylcholine is excessively decomposed by the active esterases. Therefore, the inhibitors of acetylcholinesterase (AChE) are carefully studied, as they sustain the signal transmission between the neuronal synapses and limit the occurrence of etiological factors for dementia [3].

Ginger or, *Zingiber officinale* Roscoe belongs to the Zingiberaceae botanical family and is widely used for culinary purposes, as an additive in beverages, ready meals, or bakery products. Ginger rhizomes have also been commonly used in folk medicine for thousands of years. Secondary metabolites present in its rhizomes are responsible for a number of pharmacological activities and applications, as the plant is effective in alleviating nausea, vomiting, rheumatism, inflammatory diseases of the nervous system, dyspepsia, loss of appetite, constipation, indigestion, and pain [4,5]. Moreover, the extracts of ginger are claimed to have therapeutic effects against central nervous system diseases including psychiatric disorders such as neurosis, depression, stroke, brain tumors, and Alzheimer’s disease [6]. The main group of ginger secondary metabolites are terpenoids. Among them monoterpernoids and sesquiterpenoids are highly represented. They are responsible for the rhizome’s fragrance. The following major group of compounds identified in its extract is composed of phenolics—the derivatives of ferulic acid, namely gingerols and shogaols, that affect its spicy taste.

Surprisingly, despite the huge popularity of *Zingiber officinale,* with several studies on its application in pharmacology, healthcare, and dietetics, the data on the nervous system targeting activity of single molecules from its extracts are still insufficient. Previous studies have mentioned a marked potential of ginger extracts or fractions as AChE inhibitors. The performed tests in most of the cases use an in vitro AChE inhibition assay (Ellmann’s method), that allows the calculation of the IC50 value of the tested samples in relation to other reference compounds (e.g., galanthamine or berberine) with confirmed AChE inhibitory properties. However, the activity determined in the Ellmann’s test concerns the entire tested sample. So, based on its results, it is impossible to identify the activity of single components present in the tested mixture of compounds, that are responsible for the studied properties [7].

According to Tung and co-investigators, [8] the highest AChE-inhibitory activity was calculated for the ethyl acetate fraction obtained from a crude extract of *Zingiber officinale*. Also, the manuscript by Fathy and colleagues described weak activity of phenolic constituents of ginger extracts and a moderate potential of ginger essential oil [9]. Based on these results, the authors found it interesting to study less polar constituents of ginger extracts. In the light of these findings the TLC-based bioautography screening was performed on the unpolar extracts from ginger rhizomes to identify the acetylcholinesterase inhibitors among terpens that are present in a rich mixture of secondary metabolites that are produced by ginger. The determination of their potency in comparison with galantamine—a phenanthrene alkaloid commonly administered in Alzheimer’s disease—was also achieved.

TLC bioautography is a high-throughput screening technique, which joins the application of thin layer chromatography with biodetection [7]. The applied planar method shows the composition of the studied samples and enables a clear presentation of results in the form of a picture. The technique is cheap, does not demand a high expenditure of solvents, and may analyze several samples together, in the same conditions to provide a clear picture of differences between them.

The results of the in vitro AChE inhibition studies performed by other authors have been confirmed by several in vivo assays, that determine the actual activity of ginger extracts or fractions in living organisms. Sutalangka and Wattanathorn [1] observed in their study on rats that after an oral administration of *Zingiber officinale* and *Cyperus rotundus* extracts the cognition and memorizing processes were elevated and AChE activity decreased. These extracts were found to affect the cholinergic functions of brain and decrease neurodegeneration and oxidative stress.

Another goal of this study was to collect the qualitative and quantitative results from the gas chromatography-mass spectrometry (GC-MS) analyses, that will help to select the most optimal extraction conditions and obtain enriched extracts with an increased concentration of active metabolites. In the presented work the unipolar ginger extracts will be obtained by hydrodistillation (HD), pressurized liquid extraction or accelerated solvent extraction (ASE), shaking maceration (SM), supercritical fluid extraction (SFE), and ultrasound-assisted extraction (UAE). The obtained samples will be evaluated by GC-MS analyses to provide a clear view of the efficiency of all extraction techniques and extraction conditions.

## 2. Results and Discussion

### 2.1. Anticholinesterase Assay

The AChE inhibitory assay used in the study was elaborated by Mroczek and colleagues [10], who modified the originally developed test by Marston and co-investigators [11]. In this method 2-naphtyl acetate plays a role of a substrate and is added to the mobile phase developing the TLC chromatogram. In the process of derivatization Fast Blue B salt forms a purple diazonium dye with the acetic acid coming from the decomposition of substrate by the enzyme. In the presence of an active metabolite on a TLC plate, which is bound with the enzyme, no acetic acid production occurs and no colored background is visible. As a result, active inhibitors of AChE are visible on the TLC plate in the form of colorless or yellowish spots against a purple background [12]. According to di Giovanni and collaborators, some divergence related to the identification of enzyme inhibitor may occur between the TLC bioautographic and spectrophotometric methods. However, according to the authors, the positive results of the TLC assay were confirmed by the spectrophotometric studies in the vast majority of cases, which cannot be said of the opposite [13].

**Figure 1:**
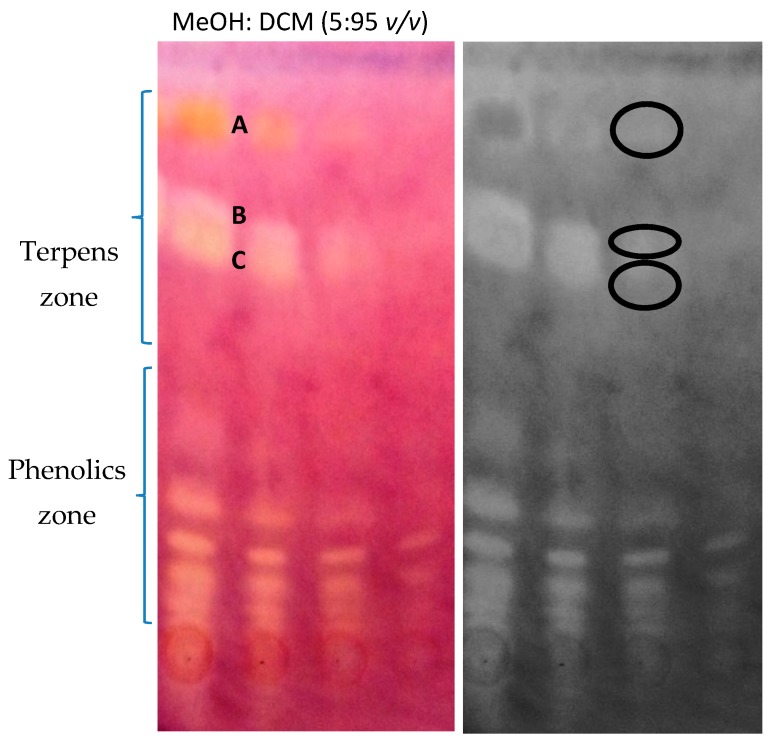
NP-TLC chromatogram presenting the ginger extract developed in the mixture of methanol and dichloromethane (5:95 *v*/*v*) after the acetylcholinesterase assay (A—*ar*-curcumene, B—α-sesquiphellandrene and C—α-zingiberene). The injection volume of the following spots, starting from the left: 3 mg/mL—15 µL, 2 mg/mL—10 µL, 1 mg/mL—5 µL, 0.5 mg/mL—2.5 µL.

Contrary to the findings of Verma and colleagues [14], who stated that essential oil from *Zingiber montanum* does not exhibit any AChE activity, in our study on *Zingiber officinale*, it was found that regions of the TLC plate (the middle third and the lower third) that contain the phenolic compounds and unpolar metabolites (the upper third) contained several active zones (Figure 1). The results of in vivo studies on rats performed by Ahmed and co-investigators [15] showed a positive physiological effect of both groups of metabolites, which were administered orally to animals in the form of an essential oil or ginger rhizomes extract at the quantity of 100 mg/kg b.w. In the same survey the Alzheimer’s disease was induced in the tested animals through aluminum chloride. Subsequent biochemical tests showed an inhibition of AChE, a decreased inflammation, and an improvement in the morphological structure of brains. Based on these findings, the herein described results are in line with the expectations.

**Figure 2:**
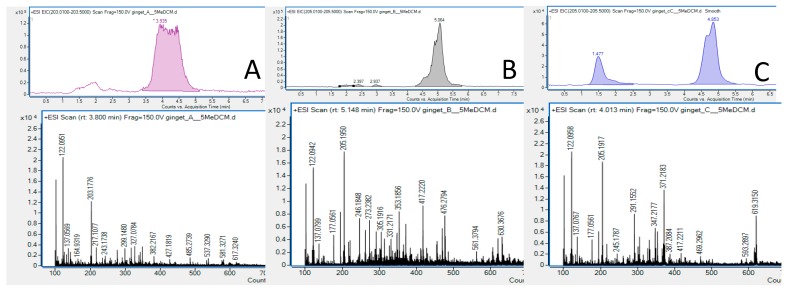
Results of TLC-HPLC-MS study of three spots that were found active in the AChE enzyme inhibition test (A–C, Figure 1) confirming the presence of: *ar-*curcumene (**A**), α-sesquiphellandrene (**B**) and *α-*zingiberene (**C**) among the major metabolites.

To accurately identify the metabolites present in the discolored zones, two TLC plates were developed at the same time in different TLC chambers, using the very same mobile phase—one with an addition of 2-naphthyl acetate, that was further sprayed with the enzyme and derivatized with Fast Blue B solution, and the other one without an addition of 2-naphthyl-acetate or any other reagents in the mobile phase. Both TLC plates were later compared with each other under the UV lamp (Camag, Muttenz, Switzerland) at two wavelengths (254 and 366 nm), and the same spots were marked across each track. A designed, unsprayed TLC plate was later subjected to TLC-HPLC-MS analysis. This technique uses a moving head, which is capable of collecting the silica gel from the TLC plate from the indicated spot and washing out the compounds present in the indicated place with the help of a mobile phase, which is used in the HPLC-MS analysis. In our study, we attached a short chromatographic column right after the TLC-MS interface, to segregate any eventually present metabolite mixtures from one another and to assess the purity of the collected spot in a better manner. The eluted components were later directed to the mass spectrometer equipped with an electrospray ionization ion source and the identity was checked using a Qualitative Navigator of the MassHunter Workstation Program (see Figure 2).

The identity of the three active zones (A, B, and C) present in the upper part of the TLC plate (terpene’s region, Figure 1) was successfully determined using a TLC-HPLC-MS approach. The volatile constituents were present in the documented mass spectra even when operating in the HPLC system, which means that they were successfully eluted out of the TLC-MS interface and a short chromatographic column by the mixture of water: acetonitrile (20:80 *v*/*v*) with the addition of 0.1% of formic acid. The analyzed mass chromatograms (Figure 2) showed significant homogenous peaks with distinct signals of *ar-*curcumene, α-sesquiphellandrene, and α-zingiberene. The identity of the last two compounds needed to be compared with the obtained GC-MS spectra, as both of them are characterized by the same molecular weight.

The potency of the three inhibitors identified in the TLC bioautography was later determined with help of an image processing software, and in relation to galantamine, which is currently a commonly used plant derived drug registered for the treatment of Alzheimer’s disease. Both galantamine and the three candidates exhibit the same mechanism of action—they are all acetylcholinesterase inhibitors.

ImageJ program used for the transformation of data provided chromatograms, that were corresponding to the whole length of a TLC track, with distinct peaks coming from the zones of AChE inhibition (Figure S1, Supplementary File). The broader and brighter the zones on the TLC plates, the higher peak areas were calculated by the program.

The calibration curve, that was obtained for galantamine in the same conditions of TLC bioautography assay, that was performed for the total extract from ginger rhizomes, helped to present the potency of the three terpenes, measured in galantamine equivalents. For the extract concentration of 2 mg/mL and the injection of 10 µL of the extract on the TLC plate the following values were obtained: for *ar-*curcumene: 0.24, for α-sesquiphellandrene: 0.28, and for α-zingiberene: 0.21 galantamine equivalents [ng]. The calibration curve equation of galantamine was equal to y = 1541.5x + 6240.5, and the *R^2^* value was calculated as 0.997.

Further results on the optimization of extraction, presented below, showed that α-zingiberene was present in much smaller quantity than the other two compounds. The SFE extract contained three times less α-sesquiphellandrene or *ar-*curcumene than α-zingiberene in the extract. Based on these calculations the latter compound shows the weakest inhibitory potential among the three metabolites.

The activity of the three terpenes in the total extract was measurable in the track, that was obtained after the introduction of 2.5 µL of a 0.5 mg/mL solution, that is equal to 1.25 µg of the total extract per spot.

The main idea of the study was to find the optimal extraction conditions that would recover the highest possible concentration of the active components from the plant matrix.

### 2.2. The Influence of the Extraction Methods on the Composition of the Volatile Extracts

#### 2.2.1. Optimization of Extraction Conditions

Owing to numerous limitations of conventional methods, some alternative techniques of extraction of volatiles were applied in this study, in the hope that they could drastically reduce the consumption of solvents and the extraction time, and at the same time increase the efficiency of the recovery process and provide the final samples that would be more rich in the three identified active molecules: *ar-*curcumene, α-sesquiphellandrene, and *α-*zingiberene.

After HD, that is usually used as the first choice, pharmacopoeial technique for the extraction of essential oils [16], SM, ASE, UAE, and SFE with carbon dioxide were used in the scheduled tests.

The authors were specifically interested in the quantitative analysis performed on ASE and SFE extracts. The former is a new extraction technique by a liquid solvent under conditions of elevated temperature and pressure. The flexibility to set various operational parameters like temperature, pressure, number of cycles, rinsing length, and volume, allows users to obtain a high-quality extract [17,18].

The application of the SFE extraction that uses CO_2_ in its supercritical state, which occurs under the precisely set temperature and pressure conditions, can produce high-quality extracts according to the rules of green chemistry, as CO_2_ turns to gas owing to the atmospheric pressure. Due to high loading capacities, low critical temperature, and pressure values, the SFE is especially used for extracting unipolar constituents from the plant matrix [19].

On the other hand, ultrasound-assisted extraction can provide good interactions and contact of the solvents with plant materials with respect to maceration, which can enhance the transfer of active compounds from a destructed cell wall [20].

The components of ginger unipolar extracts were identified in the GC–MS study by comparing the obtained data with those available in the scientific literature, their retention indices, and their mass spectra that were in turn compared with a computer library’s data or with authentic compounds. In this work, more than 20 ginger terpenes have been identified (see Table S1 in the supplementary file). The following terpenes were found in the highest quantity: α-zingiberene, α-farnesen, β-sesquiphellandrene, neral, and zingiberone.

A detailed study of the currently available scientific literature shows that the composition of terpene fraction obtained from ginger rhizomes may vary depending on the conditions in which the plant grows. The percentage composition presented in previously published data is presented in Table 1 and set together with the herein elaborated best results. As shown in Table 1, a similar tendency can be observed among the presented results.

#### 2.2.2. Quantitative Determination of Volatile Constituents in the Obtained Extracts

The results of the quantitative GC-MS analysis of ginger oleoresin after HD, ASE, UAE, SM, and SFE were obtained based on the calculated calibration curve equations of each reference compound (the obtained R^2^ values were higher than 0.99 for each curve), and are presented in Table 2 and Table S1 (supplementary material file). Classical hydrodistillation and SFE extraction were the only techniques from the tested ones that extracted simple terpene hydrocarbons, such as α- and β-pinene, myrcene, eucalyptol, camphene or others (see Table S1). The other performed extraction protocols were not efficient enough to recover this group of metabolites from the plant cells.

Concerning sesquiterpenes, which have been found to be the most active in the AChE TLC-based bioautography assay, we noted that the most effective technique that provided the highest recovery of these metabolites was SFE. Both the applied conditions delivered ca. 5 times more of the active molecules than the HD, which has been perceived as the second most effective technique. This significant difference may be affected by a very high penetrability of carbon dioxide through the plant cells. SM applied for 10 min and UAE at 30 °C within 5, 10, or 15 min recovered almost half of what HD could be able to extract. It is worth noting that the UTP extraction for 5 min gave results comparable to those of the HD. Surprisingly, ASE in all tested temperature settings (40, 60, and 80 °C) was found to be inefficient, probably due to the decomposition of volatiles occurring during the extraction process. The ASE extracts produced at 40 °C did not contain any terpenes, whereas when higher temperatures were applied, their content was still very low (see Table 2).

Concerning the compounds of interest, the SFE extraction, followed by the HD, were the techniques that recovered the highest quantity of all three active molecules. However, the former technique was able to extract above three times more of each terpene in comparison with the HD. A-Zingiberene was the most abundant among the components (Table 2). The SFE extracts operated under 300 bar delivered as much as 1.67 ± 0.11 percent of α-zingiberene, 0.51 ± 0.025 of *ar*-curcumene and 0.77 ± 0.045 of α-sesquiphellandrene from the extract. An increase in the applied operating pressure elevated the content of the studied compounds, but not significantly, as it has been expected based on the previous results [26]. On the other hand, the final percentage content of the same compound in the essential oil was calculated as 0.32 ± 0.025. In case of the three selected components of the rhizomes, the ultrasonic extraction was the most efficient, when performed shortly. Longer extraction time (10 or 15 min) decreased the content of the volatiles, and a three-cycle extraction run provided the weakest recoveries. Also, an elevated temperature had an impact on the content of terpenes in UAE. The higher the temperature applied, the lower the content of terpenes. This conclusion stays in accordance with the previously published data [27].

On the other hand, ASE required higher operation temperatures. The lowest values applied (40 °C) did not recover any terpenes from the matrix. Only some of them were present in the extracts prepared at 60 °C, whereas in the case of ASE80 samples a higher variety of terpenes was noted. Further increase in the temperature led again to a decreased content of majority of compounds. Based on these observations it could be concluded, that ASE extraction is not a preferable technique for the recovery of ginger terpenes.

## 3. Materials and Methods

### 3.1. Reagents

Reagent grade solvents used for extraction and chromatographic separation on TLC plates were purchased from Avantor Performance Materials (POCH, Gliwice, Poland). The reagents for acetylcholinesterase inhibitory tests were obtained from Sigma Aldrich (St. Louis, MO, USA), namely: acetylcholineterase (AChE) from *Electrophorus electricus* (liophylized powder type VI-S, 200-600 units per 1 mg of protein), Fast Blue B Salt, bovine serum and 2-naphtyl acetate. Similarly, the reference compounds (bornyl acetate and 1,8-cineole with a purity exceeding 95%) used for the quantitative determination of volatile constituents of the extracts were purchased from Sigma Aldrich. TLC silica-gel-covered aluminium plates (NP, F_254_) were provided by Merck (Darmstadt, Germany). Spectrometry-grade solvents for LC-MS analysis of TLC spots (acetonitrile, water, and formic acid) were manufactured by J.T. Baker (Gross-Gerau, Germany).

### 3.2. Plant Material

Fresh ginger rhizomes (500 g) were purchased from a local market in Lublin, Poland. The plant material was finely cut, thoroughly mixed and directly exposed to the tests. Only the SFE extraction was performed from the dried plant material, which was dried at a temperature of 35 °C in a laminar flow herb dryer. After the extraction, the extracts were dried on a rotary evaporator at a temperature of 35 °C and the dry residue was refrigerated at 4 °C. A voucher specimen is stored in the freezer of the Chair and Department of Pharmacognosy, at the Medical University of Lublin.

### 3.3. Thin Layer Chromatography-Bioautography Tests

All TLC chromatograms were obtained on aluminum-covered normal phase TLC plates (Merck, Darmstadt, Germany, silica gel 60 F254). First, optimization studies on the composition of the mobile phase were performed to efficiently separate phenolic compounds from terpens on the thin layer. Finally the mixture of methanol and dichloromethane (5:95 *v/v*) was selected as the most effective one based on the derivatization with 1% sulfuric acid and UV visualization.

Several concentrations of the same extract ginger equal to 40, 20, 10, 5, 2.5, and 1.25 µg of the extract per spot were introduced using a Hamilton syringe on every TLC plate. Also, the solution of galantamine—a phenanthrene alkaloid of natural origin that is commonly administered in Alzheimer’s patients —was prepared and different volumes were spotted on a clean TLC plate to enable the calculation of galantamine calibration curve, namely: 27, 18, 9, 4.5, 2, and 0.25 ng of galantamine per spot (see Figure S2 in the Supplementary File).

A revised test for the determination of AChE inhibitory properties authored by Mroczek [10] was implemented in this study on diethyl ether extracts from fresh finely cut ginger rhizome and on the galantamine solution containing plate. The total extract was obtained from the ginger rhizomes that, finely cut, were transferred to a mortar and mixed vigorously with diethyl ether using a pistel. The obtained extract was filtered through a glass Pasteur pipette filled with silica gel to remove the water. Then, it was air-dried and used for the bioactivity studies.

The TLC plates were developed in the mobile phase composed of 5% of MeOH in DCM, with an addition of 2-naphthyl acetate (30 mg for 20 mL of the mobile phase). The TLC plates of the extract and galantamine standard, which were prepared in triplicate, were evaporated to dryness and derivatized with a solution of AChE (3 units/mL) in Tris buffer (pH of 7.8), that was stabilized with bovine serum. The sprayed TLC plates were incubated for 20 min at 37 °C in high humidity conditions and after air-drying, they were visualized with a Fast Blue B salt solution (0.615 mg/mL). Separately, similar TLC plates were prepared without the derivatization, to be analyzed by TLC-LC-MS spectrometry for the identification purposes, as described in the Section 3.5.

### 3.4. Image Processing

The imaging studies were performed with help of an open source image processing program ImageJ (v.1.48). It was used for the quantitative visualization of the intensity of inhibition spots in the obtained chromatograms of the total extract and galantamine reference solution after the above described TLC bioautography assay. The developed TLC plated were dried in the air and photographed. The obtained JPG files were modified according to the previously published procedure by Olech and co-investigators [28]. The images were transformed to 8-bit colorless pictures to better expose the contrast between the background and bright areas of inhibition. The median filter was set at 5 pixels and the FFT Bandpass filter was adjusted to 40 pixels. Rectangular selection tool setting was chosen to generate the tracks’ profile lines with ‘plot lines’ setting. With help of ‘straight line selection tool’ the baseline was corrected, and the tracks were presented in the forms of chromatograms with integrated peaks (‘wand tools’ settings) coming from the zones of active inhibitors.

The obtained peak areas of the inhibition zones corresponding to the three active terpens present in the total extract were later used for the calculation of galantamine equivalents, in consideration of the galantamine calibration curve (Figures S1 and S2 in the Supplementary File).

### 3.5. Thin Layer Chromatography–High-Performance Liquid Chromatography–Mass Spectrometry Analysis

A thin layer chromatography-high-performance liquid chromatography–mass spectrometry (TLC-HPLC-ESI-TOF-MS) analysis was performed to assess the identity of the active constituents of the extracts using a mass spectrometer. The analysis was performed on plain TLC plates with zero derivatization. For this purpose, an Agilent G3250AA HPLC 1200 Series/6210 MSD TOF platform with a connected TLC-MS interface (Camag, Muttenz, Switzerland) and a short chromatographic column (Zorbax RP-18 Rapid Resolution 50 × 2.1 mm, d_p_ = 5 µm, Agilent Technologies, Santa Clara, CA USA) were used in accordance with the previously published methodology [12]. An isocratic run was selected on the HPLC chromatograph. The mobile phase was composed of solvent A (water with 0.1% formic acid) and solvent B (acetonitrile with 0.1% formic acid), that were pumped in the ratio of 20:80 (*v*/*v*). The total analysis time was set at 5 min from the collection of the spots’ content by a TLC interface (Camag, Muttenz, Switzerland). The application time was set at 3 s. The following settings of the mass spectrometer were applied: positive ionization mode, gas and vaporizer temperatures 350 °C, drying gas flow 10 L/min, nebulizer 30 psig, fragmentor voltage 175 V, skimmer voltage 65 V, and capillary voltage 2000 V. MassHunter Workstation program (version B.02.00) was used to handle the obtained data.

The TLC-MS interface was also used for the identity determination studies of the active components to directly collect the constituents of the active spots in a vial. Owing to this, the collected metabolites could be later analyzed by GC-MS spectrometry and identified by the included spectral libraries.

### 3.6. Extraction Conditions

As described below, several extraction techniques were applied to optimize the recovery of the selected terpenes out of the plant matrix. Table 3 lists all applied conditions.

#### 3.6.1. Hydrodistillation in a Deryng Apparatus

Finely cut ginger rhizomes (50 g) were placed in a round-bottomed flask and 130 mL of distilled water was added. The hydrodistillation process was performed 3 h long in a Deryng apparatus. After this duration, the obtained oil was mixed with n-hexane, dried from water over silica gel, and stored in a sealed vial at a low temperature (4 °C) before the GC-MS analysis.

#### 3.6.2. Accelerated Solvent Extraction (ASE)

Finely cut rhizomes (1 g) were placed in a stainless steel extraction cell and extracted with n-hexane at the following temperatures: 40, 60, and 80 °C. Each extraction was performed three times. The other working conditions of the ASE 100 extractor (Dionex, Sunnyvale, CA, USA) were set as follows: Static time 5 min, flush volume 60%, purge time 20 s, and number of extraction cycles 3. The pressure was maintained at ca. 100 bar throughout the extraction process. The obtained extracts were first evaporated on a rotary vacuum evaporator at a temperature of 35 °C and then directly dissolved in n-hexane before the GC-MS analysis.

#### 3.6.3. Ultrasound Assisted Extraction (UAE)

Portions of freshly cut ginger rhizomes (1 g) were placed in twelve Eppendorf tubes and extracted with 1.2 mL of dichloromethane under the following conditions: i) 4 Eppendorf tubes at room temperature, ii) four tubes at 30 °C, and iii) the last four tubes at 60 °C. The sonication process of the ginger rhizomes was conducted at different time intervals of 5, 10, 15 min and 3 × 5 min, in triplicate. After the extraction, the vials were centrifuged, and the supernatants were transferred to an amber glass vial and evaporated to dryness at 30 °C with the help an Eppendorf Concentrator Plus (Hamburg, Germany). The dried extracts were refrigerated and dissolved in *n*-hexane at the concentration of 10 mg/mL before to the GC-MS analysis.

#### 3.6.4. Shaking Maceration

Finely cut ginger rhizomes (1 g) were placed in Eppendorf tubes and extracted with 1.2 mL of dichloromethane on a shaking platform (300 rpm) for 5, 10, 15 min and 3 × 5 min, in triplicate. After each extraction, the vials were centrifuged at 1500 rpm and the supernatants were collected in fresh vials, evaporated to dryness using an Eppendorf Concentrator Plus centrifuge, and stored at 4 °C. Before the GC-MS analysis, they were re-dissolved in *n*-hexane at a concentration of 10 mg/mL.

#### 3.6.5. Supercritical Fluid Extraction (SFE)

Ginger was sent to INS in the form of cut pieces of about 2 cm in size. These pieces were then ground on a Retsch mill (model SM100) using a 1.5 mm sieve. The raw material prepared in this way was extracted through the SITEC/Switzerland research plant. The test plant parameters were as follows: The capacity of the extractor basket was 0.6 dm^3^, the maximum working pressure was 100 MPa, and the maximum working temperature was 200 °C. The experiments were carried out in duplicate at 115 bar and 300 bar pressure. Other parameters such as temperature, extraction time, and flow rate were kept constant. The average CO_2_ flow rate was 5.46 kg/h. The operating parameters of the research plant are summarized in Table 4. In contrast to other techniques evaluated in this study, the standard deviation values obtained for this type of extraction come from a triple injection of the same extracts.

### 3.7. GC-MS Analysis of Essential Oil and Volatile Extracts

The GC–MS analysis was performed with a Shimadzu GC-2010 Plus GC instrument coupled with a Shimadzu QP2010 Ultra mass spectrometer (Kyoto, Japan). Compounds were separated on a fused-silica capillary column ZB-5 MS (30 m, 0.25 mm i.d.) with a film thickness of 0.25 μm (Phenomenex, Torrrance, CA, USA). The following oven temperature program was initiated at 50 °C, held for 3 min, then increased to 250 °C at the rate of 5 °C/min, and held for 15 min. The spectrometers were operated in the electron impact mode, the scan range was 40–500 amu, the ionization energy was 70 eV, and the scan rate was 0.20 s per scan. The injector, interface, and ion source were kept at 280, 250, and 220 °C, respectively. A split injection was performed with a split ratio of 1:20 and helium was used as a carrier gas of 1.0 mL/min flow rate. The retention indices were determined in relation to a homologous series of *n*-alkanes (C8–C24) under the same operating conditions. Compounds were identified using a computer-assisted spectral library (MassFinder 2.1; NIST 2011).

The quantitative analysis of terpenes was performed based on the calibration curves of bornyl acetate and 1, 8-cineol. Both the compounds were diluted in hexane at the concentration of 1 mg/mL. Out of this stock solution, six dilutions were prepared for each compound to construct a calibration curve equation, namely: 0.5, 0.1, 0.05, 0.025, 0.01, 0.005 mg/mL. The injection volume was set at 2 µL.

## 4. Conclusions

The TLC-bioautography technique that has been used in the survey to identify the potentially active AChE inhibitors from the ginger rhizomes has been found effective enough to fish out the drug candidates and, coupled with TLC-HPLC-MS approach, to identify the active metabolites with no need for their isolation from the mixture. The optimization of extraction, that has been performed for the sake of a better recovery of the identified three active components: *ar*-curcumene, α-sesquiphellandrene, and α-zingiberene showed the best efficiency of the HD, SFE and SM 10.

## Figures and Tables

**Table 1:** Comparison of the literature data and the obtained results concerning the percentage content of volatile extracts from ginger rhizomes.

References	[21]	[22]		[23]	[24]	[17]		[25]	Our Results				
Extraction Method	MD–SPME	M in Water	M in Sesame Oil (1:5)	HD in Clevenger	M in Diethyl Ether	HD	SFE 250bar, 60 °C	HD	HD	SM10	UAE60 10	ASE 80	SFE 115bar, 40 °C
α-zingiberene	15.48	30.06	29.35	30.81	37.9	1.64	19.77		27.24	14.11	14.48	18.11	32.89
α-farnesen		9.75	9.26		9.6	1.29	6.31		5.27	13.59	12.59	9.5	14.64
*ar*-curcumene		5.18	5.33		6.3	11.32	6.12		2.77	5.78	4.73	2.75	8.76
camphene		7.69				4.88		11.52	7.13				2.99
β-bisabolene		6.53	5.83			4.45	4.52		1.65	3.41	3.66	2.54	6.74
ɣ-curcumene		5.9	5.62										
myrcene				4.6				3.04	1.57				0.84
1,8-cineole				3.9		3.14		23.88	9.74				7.63
α-pinene				3.6		1.33		3.31	2.23				1.21
neral				3.9					7.36	8.98	7.7	13.4	8.98
geranial	5.25				8.2	10.66	3.85	14.19					
β-sesquiphellandrene	5.54	10.71	9.6		11.4	0.74	10.9		3.28	9.36	9.36	7.57	
α-phellandrene	22.84	13.51	8.87	2.8					0.21			0.45	0.2
γ-Terpinene				2.5	5.1								
β-pinene				0.74					0.37				

M—maceration, HD—hydrodistillation, SM—shaking maceration, UAE60 10—ultrasound assisted extraction at 60 °C and 10 min, ASE 80—accelerated solvent extraction at 80 °C, 3 times 5 min, SFE—supercritical fluid extraction, SPME—solid phase microextraction; The colors were obtained from Excel table processing—conditional formatting—and show the quantitative differences between the values; green background highlights the highest quantity, the red color underlines the smallest concentration presented.

**Table 2:** Quantitative determination of the major constituents of volatile extracts in µg/g of extract.

	HD	SE 5	SE 10	SE 15	SE 3 × 5	UAE 5	UAE 10	UAE 15	UAE 3 × 5	UAE 30 5	UAE 30 10	UAE 30 15	UAE 30 3 × 5	UAE 60 5	UAE 60 10	UAE 60 15	UAE 60 3 × 5	ASE 40	ASE 60	ASE 80	SFE 1-2	SFE 3-4
α-Terpineol	56.89 ± 1.8	7.24 ± 3.4	13.92 ± 1.0	12.45 ± 8.7	7.33 ± 1.9	14.64 ± 8.4	17.05 ± 4.0	11.95 ± 2.04	7.72 ± 3.8	19.53 ± 4.2	15.13 ± 3.2	19.40 ± 4.6	64.70 ± 1.0	14.31 ± 5.6	17.26 ± 3.7	20.05 ± 5.1	0.00	0.00	0.00	0.00	35.16 ± 2.6	3.38 ± 2.9
Citronellol	57.58 ± 2.9	7.68 ± 1.1	19.02 ± 3.4	14.62 ± 13.7	4.88 ± 4.2	20.47 ± 9.5	18.62 ± 5.2	14.87 ± 3.8	8.12 ± 2.0	22.65 ± 8.7	14.79 ± 2.7	21.76 ± 2.3	6.34 ± 1.7	16.26 ± 3.3	17.90 ± 9.0	19.23 ± 5.7	3.49 ± 7.8	0.00	0.00	29.52 ± 2.6	37.13 ± 2.6	32.80 ± 3.5
Neral	625.07 ± 43.1	18.57 ±.9	48.01 ± 5.2	21.17 ± 2.9	15.21 ± 2.1	29.01 ± 7.8	39.15 ± 7.1	29.40 ± 2.8	13.33 ± 2.2	51.83 ± 11.7	36.25 ± 5.1	34.09 ± 8.3	16.74 ± 6.8	31.61 ± 4.4	43.19 ± 3.1	58.52 ± 3.8	5.55 ± 2.9	0.00	0.00	12.74 ± 0.6	234.42 ± 19.5	207.80 ± 31.2
Geraniol	173.94 ± 12.0	47.83 ± 4.3	73.72 ± 5.1	59.61 ± 4.2	27.73 ± 2.1	139.62 ± 19.7	119.03 ± 21.0	90.32 ± 8.4	50.44 ± 5.0	127.15 ± 13.8	81.02 ± 11.4	113.88 ± 15.1	38.38 ± 4.3	115.36 ± 9.1	112.87 ± 5.8	100.21 ± 12.9	30.67 ± 3.3	0.00	80.03 ± 0.8	206.60 ± 7.0	218.70 ± 18.3	23.03 ± 2.5
***ar*-Curcumene**	**120.95 ± 3.6**	**17.9 ± 1.52**	**59.53 ± 3.26**	**45.98 ± 11.7**	**21.81 ±1.5**	**105.47 ± 16.2**	**59.31 ± 1.0**	**47.64 ± 11.5**	**33.61 ± 2.6**	**69.99 ± 8.4**	**69.50 ± 5.3**	**60.50 ± 2.21**	**24.12 ± 2.61**	**41.09 ± 1.2**	**46.06 ± 7.5**	**49.25 ± 3.5**	**5.92 ± 1.2**	**0.00**	**45.85 ± 4.2**	**35.84 ± 7.3**	**433.62 ± 42.2**	**511.50 ± 25.8**
**α-Zingiberene**	**320.82 ± 25.4**	**554.40 ± 11.5**	**145.43 ± 21.4**	**85.7 ± 3.2**	**57.64 ± 4.1**	**333.29 ± 29.1**	**164.88 ± 13.4**	**112.80 ± 19.3**	**73.78 ± 8.57**	**170.30 ± 21.1**	**146.30 ± 4.5**	**169.02 ± 5.07**	**18.87 ± 5.4**	**125.79 ± 2.86**	**124.65 ± 15.0**	**117.6 ± 13.9**	**9.93 ± 6.71**	**0.00**	**0.00**	**110.90 ± 6.9**	**1627.40 ± 120.6**	**1670.90 ± 111.0**
**α-Sesquiphel-landrene**	**143.07 ± 35.1**	**49.43 ±7.2**	**96.48 ± 10.3**	**71.04 ±5.7**	**34.70 ±2.2**	**197.20 ± 11.6**	**130.08 ± 11.7**	**88.70 ± 9.3**	**46.48 ± 6.2**	**137.90 ± 10.7**	**106.28 ±12.9**	**152.65 ± 15.6**	**32.44 ± 4.5**	**81.33 ± 1.2**	**90.00 ± 19.2**	**95.15 ± 11.0**	**5.28 ± 3.6**	**0.00**	**0.00**	**98.86 ± 15.2**	**458** **.70 ± 38.2**	**769** **.40 ±42.2**

The colors were obtained from Excel conditional formatting of Table data and show the quantitative differences between the values; green background: the highest quantity, the red color: the smallest concentration presented.

**Table 3:** The list of the obtained extracts (atm—amospheric pressure, rt—room temperature).

Code.	Extraction Technique	Pressure [bar]	Temperature [°C]	Extraction Time [min]	Pressure [bar]	Extractant	Remarks
SFE 1-2	Supercritical fluid extraction	115	40	240	115	CO_2_	Liquid oil extract, yellow
SFE 3-4	Supercritical fluid extraction	300	40	240	300	CO_2_	Thick oil extract, yellow.
HD	Deryng apparatus hydrodistillation	atm	>100	180	atm	H_2_O	Liquid oil extract, light yellow
SM5	Shaking extraction	atm	rt	5	atm	CH_2_Cl_2_	Liquid extract, yellow
SM10	Shaking extraction	atm	rt	10	atm	CH_2_Cl_2_	Liquid extract, yellow
SM15	Shaking extraction	atm	rt	15	atm	CH_2_Cl_2_	Liquid extract, yellow
SM 3 × 5	Shaking extraction	atm	rt	3 × 5 min	atm	CH_2_Cl_2_	Liquid extract, yellow
UAE 5	Ultrasound-assisted extraction	atm	rt	5	atm	CH_2_Cl_2_	Liquid extract, yellow
UAE 10	Ultrasound-assisted extraction	atm	rt	10	atm	CH_2_Cl_2_	Liquid extract, yellow
UAE 15	Ultrasound-assisted extraction	atm	rt	15	atm	CH_2_Cl_2_	Liquid extract, yellow
UAE 3 × 5	Ultrasound-assisted extraction	atm	rt	3 × 5 min	atm	CH_2_Cl_2_	Liquid extract, yellow
UAE30 5	Ultrasound-assisted extraction	atm	30	5	atm	CH_2_Cl_2_	Liquid extract, yellow
UAE30 10	Ultrasound-assisted extraction	atm	30	10	atm	CH_2_Cl_2_	Liquid extract, yellow
UAE30 15	Ultrasound-assisted extraction	atm	30	15	atm	CH_2_Cl_2_	Liquid extract, yellow
UAE30 3 × 5	Ultrasound-assisted extraction	atm	30	3 × 5 min	atm	CH_2_Cl_2_	Liquid extract, yellow
UAE60 5	Ultrasound-assisted extraction	atm	60	5	atm	CH_2_Cl_2_	Liquid extract, yellow
UAE60 10	Ultrasound-assisted extraction	atm	60	10	atm	CH_2_Cl_2_	Liquid extract, yellow
UAE60 15	Ultrasound-assisted extraction	atm	60	15	atm	CH_2_Cl_2_	Liquid extract, yellow
UAE60 3 × 5	Ultrasound-assisted extraction	atm	60	3 × 5 min	atm	CH_2_Cl_2_	Liquid extract, yellow
ASE 40	Accelerated solvent extraction	100	40	3 × 5 min	100	*n*-hexane	Liquid extract, dark yellow
ASE 60	Accelerated solvent extraction	100	60	3 × 5 min	100	*n*-hexane	Liquid extract, dark yellow
ASE 80	Accelerated solvent extraction	100	80	3 × 5 min	100	*n*-hexane	Liquid extract, dark yellow

**Table 4:** Supercritical fluid extraction (SFE) extractor operating parameters.

No.	Batch [g]	Pressure [bar]	Temperature [°C]	Extraction Time [min]	Yield [%mass]	Remarks
1	200.04	115	40	240	2.50	Liquid oil extract, yellow
2	200.75	300	40	240	3.09	Thick oil extract, yellow.

## Supplementary Materials

Supplementary Materials are available online.
