# Supplementary material for: Acetylcholinesterase Inhibitors among Zingiber officinale Terpenes—Extraction Conditions and Thin Layer Chromatography-Based Bioautography Studies
Source: Molecules. 2020 Apr 3;25(7):1643. doi: 10.3390/molecules25071643 (PMC7181192; doi:10.3390/molecules25071643)
Supplement: Supplementary file 1 [file molecules-25-01643-s001.pdf]

molecules

MDPI

SUPPLEMENTARY FILE

Article

Acetylcholinesterase inhibitors among *Zingiber officinale* terpenes – extraction conditions and thin layer chromatography-based bioautography studies

Lidia Czernicka [1], Agnieszka Ludwiczuk [2], Edward Rój [3], Zbigniew Marzec [1], Agata Jarząb [5] and Wirginia Kukula-Koch [6,*]

[1] Chair and Department of Food and Nutrition, Medical University of Lublin, 4a Chodźki Str., 20-093 Lublin, Poland; lidia.czernicka@umlub.pl (L.C.); zbigniew.marzec@umlub.pl (Z.M.)
[2] Independent Laboratory of Natural Products Chemistry, Chair and Department of Pharmacognosy, Medical University of Lublin; 1 Chodzki Str., 20-093 Lublin, Poland; aludwiczuk@pharmacognosy.org
[3] Supercritical Extraction Department, ŁUKASIEWICZ Research Network—New Chemical Syntheses Institute, Tysiąclecia Państwa Polskiego Ave. 13a, 24-110 Puławy, Poland; edward.roj@ins.pulawy.pl
[4] Department of Biochemistry and Molecular Biology, Medical University of Lublin, 1 Chodzki St., 20-093 Lublin, Poland; agata.jarzab@umlub.pl
[5] Chair and Department of Pharmacognosy, Medical University of Lublin; 1 Chodzki Str., 20-093 Lublin, Poland
* Correspondence: virginia.kukula@gmail.com; Tel.: +488-1448-7087

Table S1. The comparison of the monoterpene content of essential oil obtained in Deryng apparatus with supercritical extracts (SFE1-2 and SFE 3-4).

Volatile compound	RT	RI	HD. [mg/g]	SFE 1-2 [mg/g]	SFE 3-4 [mg/g]
α-Pinene	7.871	948	0.09716	0.05547	0.01128
Camphene	8.387	943	0.31088	0.14799	0.03264
β-Pinene	9.251	943	0.01634	0.00000	0.00000
Myrcene	9.624	958	0.06841	0.04168	0.00000
Octanal	10.045	1005	0.00814	0.00000	0.00000
α-Phellandrene	10.159	969	0.00918	0.01002	0.06286
p-Cymene	10.751	1042	0.00402	0.00000	0.00000
Limonene	10.892	1018	0.05062	0.00000	0.00000
1,8-Cineole	10.979	1059	0.42480	0.37746	0.05320
2-Carene	12.666	948	0.01170	0.00805	0.00223
Linalool	13.233	1082	0.01899	0.02186	0.00000
Citronellal	14.724	1125	0.02254	0.00598	0.00475
Borneol	15.429	1138	0.05605	0.02845	0.02999
Terpinen-4-ol	15.649	1137	0.00960	0.00567	0.00456

Table S2. The percentage content of volatile constituents of UAE60 15 Extract based on the GC-MS analysis results.

	Ret. time	Area	Area %	Name
1.	15.395	232050	0.25	Borneol
2.	16.081	625743	0.67	L-α-terpineol
3.	16.270	141967	0.15	Decanal
4.	16.315	600124	0.15	Citronellol
5.	17.634	3127510	3.36	Geraniol
6.	18.102	4402376	4.73	Neral
7.	20.516	468769	0.50	Geranic acid
8.	21.079	188557	0.20	Geranyl acetate
9.	21.407	101997	0.11	β-elemene
10.	23.788	1537258	1.65	Ar-curcumene
11.	24.131	3670754	3.94	α-Zingiberene
12.	24.333	3539512	3.80	α - farnesen
13.	24.451	935904	1.00	β - bisabolen
14.	24.716	365442	0.39	cubedol
15	24.847	2969597	3.19	β-sesquiphellandrene
16.	25.570	589069	0.63	Elemol
17.	25.673	690833	0.74	Nerolidol
18.	26.427	400149	0.43	7-epi-cis-sesquisabinene
19.	26.987	647569	0.70	α-bisabolol
20.	27.203	269399	0.29	Germacrene
21.	27.607	2241031	2.41	Zingiberone
